# Resolutions of Respect

**Published:** 1897-07

**Authors:** 


### RESOLUTIONS OF RESPECT.—DR. FRANK ABBOTT.

At a special meeting of the Alumni Association of the New York College of Dentistry, held April 29, 1897, the following preamble and resolutions were unanimously adopted:

Whereas, In the wisdom of an all-wise Providence the Alumni Association of New York College of Dentistry is called upon to mourn the loss by death of our late professor and dean, Frank Abbott, M.D., therefore, be it

*Resolved*, That the death of our late professor and friend calls for expressions of esteem for one whose character was above reproach, whose undeviating justice and loving-kindness to all with whom he came in contact leave impressions that cannot be effaced, and whose influence for good, both in and out of his chosen profession, was immeasurable. One of the brightest lights of our profession has been extinguished, but its rays, sure and strong, will continue to illuminate while dentistry lasts.

*Resolved*, That this preamble and resolutions be placed in full upon the minutes, and that a copy suitably engrossed be presented to his bereaved family for whom we feel the sincerest sympathy.

W. D. TENISON.
B. C. WASH.
W. CHAS. GATTSCHADT, *Chairman.*

F. T. HANNEMANN,
*Secretary of A. A. N. Y. C. D.*

